# The effect of behavioral therapy on dysphagia of acute ischemic stroke patients feeding with a nasogastric tube

**DOI:** 10.1371/journal.pone.0299068

**Published:** 2024-04-18

**Authors:** Nguyen Thi Thu Hien, Tran Huu Thong, Le Thanh Tung, Tran Thi Tinh, Tran Huu Trung

**Affiliations:** 1 Dermatology Department, Bach Mai Hospital, Hanoi, Vietnam; 2 Department of Nursing, Bach Mai Hospital, Hanoi, Vietnam; 3 Center for Emergency Medicine, Bach Mai Hospital, Hanoi, Vietnam; 4 Department of Emergency and Critical Care Medicine, Hanoi Medical University, Hanoi, Vietnam; 5 Faculty of Medicine, University of Medicine and Pharmacy, Vietnam National University, Hanoi, Vietnam; 6 Nam Dinh University of Nursing, Nam Dinh, Vietnam; 7 Center of Neurology, Bach Mai Hospital, Hanoi, Vietnam; 8 Hanoi Medical University, Hanoi, Vietnam; Universita degli Studi di Roma La Sapienza, ITALY

## Abstract

This prospective observational study aimed to assess the impact of behavioral therapy on dysphagia in patients with acute ischemic stroke undergoing nasogastric tube feeding. The study was conducted between June 2020 and May 2022 at the Neurological Center of Bach Mai Hospital, Vietnam, with a sample size of 230 patients divided into two groups: a normal and a behavioral therapy group. The normal therapy group received routine care and treatment based on standard protocols, while the behavioral therapy group underwent daily swallowing exercises for approximately 60 minutes. The Gugging Swallowing Screen (GUSS) was utilized to screen individuals with dysphagia, and the difference-in-differences (DID) method was adopted to estimate the effect of behavioral therapy on dysphagia patients. The study concluded that behavioral therapy improved dysphagia in patients with acute ischemic stroke undergoing nasogastric tube feeding. This study highlights the potential of behavioral therapy as an effective intervention for dysphagia rehabilitation in stroke patients.

## Introduction

Dysphagia, or difficulty swallowing, is a common complication of acute ischemic stroke, affecting up to 78% of patients in the first few days after stroke onset [1, 2]. This can lead to severe consequences such as aspiration pneumonia, malnutrition, and dehydration, increasing mortality, morbidity, and healthcare costs [1, 3]. Dysphagia in stroke patients can increase the risk of pneumonia, which can cause severe illness and death [4]. It was reported that dysphagia could associated with poor functional recovery, post-stroke depression, and prolonged hospitalization [5]. Although dysphagia often improves spontaneously within the first month after stroke, it may persist in many patients, necessitating swallowing rehabilitation [6].

There have been various approaches to addressing dysphagia, from medications to surgeries [7–13]. However, behavioral therapy has emerged as a promising intervention [14]. This treatment involves actively and repeatedly performing exercises or maneuvers to improve swallowing function [15]. Such interventions can help prevent the aspiration of thin liquids, enhance dysphagia rehabilitation, strengthen swallowing muscles, and improve swallowing function in patients with neurogenic dysphagia and stroke-related swallowing dysfunction [14, 15]. Furthermore, studies have demonstrated that behavioral therapy can strengthen the base of the tongue to the posterior pharyngeal wall, leading to improved dysphagia outcomes [15].

The Gugging Swallowing Screen (GUSS) tool is widely utilized among medical professionals requiring swift bedside screening for dysphagia [16, 17]. Its straightforward and efficient approach to assessing dysphagia progress is well-regarded [16, 17]. Despite its popularity, there is a shortage of research on the short-term effects of using the GUSS dysphagia assessment application for behavioral therapy. Therefore, this study aimed to examine the impact of behavioral therapy on patients with acute ischemic stroke who are undergoing nasogastric tube feeding.

## Methods

### Study design and setting

A prospective observational study was conducted at the Neurological Center of Bach Mai Hospital in Vietnam, covering the period from June 2020 to May 2022. Bach Mai hospital is a tertiary hospital providing health care services to people in Hanoi and the northern provinces of Vietnam. This general hospital, including the Neurological Center, collects and treats patients with neurological diseases. Patients with acute ischemic stroke account for a large proportion of patients with neurological diseases treated at the center. Here, cases of acute ischemic stroke were confirmed by computed tomography (CT) or magnetic resonance imaging (MRI). The research proposal received approval from the research ethics committees at Nam Dinh University of Nursing under approval number 1603/GCN-HDDD, and written informed consent was obtained from each subject before recruitment.

### Study participants

The study involved individuals who were diagnosed with acute cerebral infarction and were experiencing dysphagia, necessitating the use of a nasogastric tube for nutrition. To be eligible for participation, individuals had to be 18 years or older, conscious with a Glasgow Coma Scale (GCS) score exceeding 13 points, and capable of comprehending communicative language. In addition, only patients with moderate strokes classified by the National Institutes of Health Stroke Scale (NIHSS) were included in the study. Those requiring oxygen, endotracheal intubation, or mechanical ventilation were excluded from the study, as were those with jaw or facial trauma, myocardial infarction, Wernicke’s aphasia, or dementia. In addition, individuals without teeth (crowns), a history of prior swallowing difficulties, or evidence of pre-existing pneumonia were not considered for the study. We also excluded cases with a treatment duration of less than three days.

### Sample size

We used the sample size formula to determine the contrast between the two proportions and adhered to the World Health Organization’s guidelines. We established a significance level of 0.05 and an absolute precision of 0.1 to calculate the required sample size. With a predicted dysphagia recovery rate of 97% for the behavioral therapy group and 62.5% for the normal therapy group, along with a non-response rate of 10%, we determined that a minimum of 111 patients was needed. However, to ensure a thorough analysis, we opted to include 115 patients per group, resulting in a total of 230 enrolled patients.

### Sampling

Patients who met the study criteria and consented to participate were selected from the Neurological Center. The center comprised two wards, where patients receive routine care and treatment following standard procedures and protocols. Nutritional counseling, dietary guidance, and instructions on appropriate eating positions were provided to all patients. In addition, patients from one of the wards were enrolled in a behavioral therapy group and provided with additional swallowing exercises. Hence, in this study, one group received normal therapy (normal group), while the other received normal therapy along with swallowing exercises (behavioral therapy group).

### Intervention

The behavioral therapy group participated in daily swallowing exercises that lasted around 60 minutes. These exercises included training for the tongue, palate, facial muscles, and mouth movements. Each exercise was tailored to the patient’s needs, with specific schedules and timings. The tongue exercises lasted 15 minutes, palate exercises took 5 minutes, facial exercises were given 15 minutes, maxillofacial exercises were allotted 10 minutes, and pharynx and larynx exercises were completed within 15 minutes. Tongue exercises aim to strengthen and coordinate the muscles of the tongue. Palate exercises help to improve the function and coordination of the palate muscles, which are involved in sealing off the nasal passages during swallowing to prevent food or liquid from entering the nose. Facial exercises target facial muscles, including lip movement and facial expressions. Maxillofacial exercises focus on the muscles associated with the jaw and upper face; strengthening and training these muscles might aid in chewing and the initial stages of swallowing. Pharynx and larynx exercises concentrate on muscles in the pharynx (throat) and larynx (voice box), which are pivotal for properly progressing the swallowing process. A nurse with a certified degree in speech and language therapy was responsible for instructing patients on swallowing exercises.

### Variables

In this study, the primary outcome variable was whether a patient had severe dysphagia, defined as a GUSS score between 0 and 9 [16, 18]. The GUSS was used to screen for dysphagia and consists of indirect and direct swallowing tests. The total GUSS score ranges from 0 to 20, with higher scores indicating less severe dysphagia [18].

The explanatory variables included in this study were sex, age group (<60, 60–69, > = 70), area (urban or rural), education (primary or less, secondary school, high school and above), communication disorder (yes or no), previous stroke (yes or no), hypertension (yes or no), diabetes mellitus (yes or no), and obesity (BMI≥25). Obesity was defined by calculating the patient’s BMI, which was obtained by dividing their weight by the square of their height.

### Statistical analysis

In this study, we presented qualitative data as frequencies and percentages. We used Chi-square tests to compare the differences between groups for qualitative variables. Furthermore, we employed Chi-square tests to compare the prevalence of severe dysphagia between the behavioral and normal therapy groups based on various factors. To estimate the effect of behavioral therapy, we utilized the "diff" command for difference-in-differences (DID) estimation, which is a STATA package developed by Juan M. Villa [19]. The "diff" command, which is not a native feature but is installable in STATA, estimates the treatment effect from combined baseline and subsequent data. This method hinges on comparing outcomes before and after the treatment in both groups, effectively managing unobserved variables that remain constant. By comparing outcome changes over time between a treatment group and a control group, DiD evaluates the effect of an intervention program. We also applied the kernel propensity-score DID to adjust for different factors. All statistical analyses were conducted using STATA® 14. The level of statistical significance was set at a P-value of 0.05.

## Results

**Fig 1:**
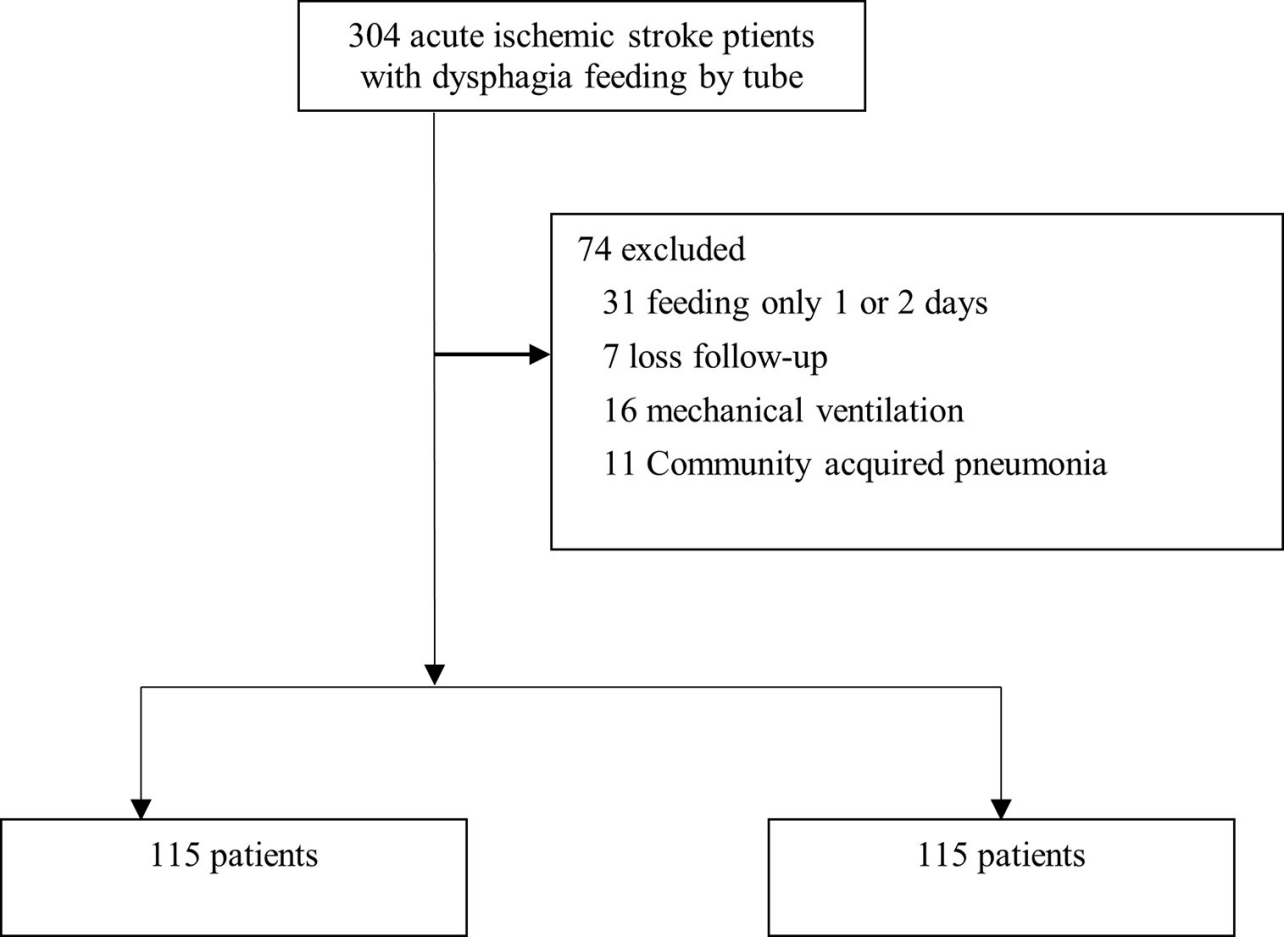
Flowchart of patients’ selection.

Of the 304 patients, 74 had to be excluded from the study for various reasons, such as being fed for only 1 or 2 days, losing follow-up, pneumonia, or mechanical ventilation. The remaining 230 patients were then divided into two groups: a behavioral therapy group and a normal therapy group (Fig 1).

The general characteristics of the study participants are presented in Table 1. Both therapy groups had a similar sex distribution, with approximately two-thirds of participants being male. In addition, the two therapy groups had no significant difference in age group distribution. Similarly, factors such as area, education, obesity, communication disorder, previous stroke, hypertension, and diabetes mellitus were not significantly different between the two therapy groups.

**Table 1:** General characteristics of study participants.

Variables	Behavioral therapy N (%)	Normal therapy N (%)	P value
Sex			
Male	77 (67.0)	73 (63.5)	0.58
Female	38 (33.0)	42 (36.5)	
Age group (year)			
<60	39 (33.9)	26 (22.6)	0.09
60–69	28 (24.4)	40 (34.8)	
≥70	48 (41.7)	49 (42.6)	
Area			
Urban	34 (29.6)	25 (21.7)	0.17
Rural	81 (70.4)	90 (78.3)	
Education			
Primary or less	38 (33.0)	30 (26.1)	0.39
Secondary school	37 (32.2)	36 (31.3)	
High school and above	40 (34.8)	49 (42.6)	
Obesity			
Yes	15 (13.0)	21 (18.3)	0.28
No	100 (87.0)	94 (81.7)	
Communication disorder			
Yes	62 (53.9)	72 (62.6)	0.18
No	53 (46.1)	43 (37.4)	
Previous stroke			
Yes	24 (20.9)	34 (29.6)	0.13
No	91 (79.1)	81 (70.4)	
Hypertension			
Yes	70 (60.9)	80 (69.6)	0.17
No	45 (39.1)	35 (30.4)	
Diabetes Mellitus			
Yes	27 (23.5)	25 (21.7)	0.75
No	88 (76.5)	90 (78.3)	

Table 2 displays the rate of severe dysphagia before and after treatment based on various factors. The prevalence of severe dysphagia before treatment was higher than after treatment for all variables, as indicated by the lower prevalence values after treatment. The difference in prevalence before and after treatment is statistically significant (all P values <0.01).

**Table 2:** The prevalence of severe dysphagia before and after intervention by different factors.

Variables	Severe dysphagia					
	Behavioral therapy			Normal therapy		
	Before	After	P value	Before	After	P value
All	85 (73.9)	3 (2.6)	<0.01	81 (70.4)	22 (19.1)	<0.01
Sex						
Male	56 (72.7)	1 (1.3)	<0.01	49 (67.1)	15 (20.6)	<0.01
Female	29 (76.3)	2 (5.3)	<0.01	32 (76.2)	7 (16.7)	<0.01
Age group (year)						
<60	24 (61.5)	1 (2.6)	<0.01	17 (65.4)	2 (7.7)	<0.01
60–69	19 (67.9)	0 (0.0)	<0.01	29 (72.5)	8 (20.0)	<0.01
≥70	42 (87.5)	2 (4.2)	<0.01	35 (71.4)	12 (24.5)	<0.01
Area						
Urban	27 (79.4)	0 (0.0)	<0.01	21 (84.0)	4 (16.0)	<0.01
Rural	58 (71.6)	3 (3.7)	<0.01	60 (66.7)	18 (20.0)	<0.01
Education						
Primary or less	31 (81.6)	1 (2.6)	<0.01	21 (70.0)	7 (23.3)	<0.01
Secondary school	26 (70.3)	1 (2.7)	<0.01	24 (66.7)	4 (11.1)	<0.01
High school and above	28 (70.0)	1 (2.5)	<0.01	36 (73.5)	11 (22.5)	<0.01
Obesity						
Yes	12 (80.0)	1 (6.7)	<0.01	15 (71.4)	4 (19.1)	<0.01
No	73 (74.0)	2 (2.0)	<0.01	66 (70.2)	18 (19.2)	<0.01
Communication disorder						
Yes	53 (85.5)	2 (3.2)	<0.01	49 (68.1)	11 (15.3)	<0.01
No	32 (60.4)	1 (1.9)	<0.01	32 (74.4)	11 (25.6)	<0.01
Previous stroke						
Yes	20 (83.3)	0 (0.0)	<0.01	25 (73.5)	8 (23.5)	<0.01
No	65 (71.4)	3 (3.3)	<0.01	56 (69.1)	14 (17.3)	<0.01
Hypertension						
Yes	54 (77.1)	2 (2.9)	<0.01	61 (76.3)	16 (20.0)	<0.01
No	31 (68.9)	1 (2.2)	<0.01	20 (57.1)	6 (17.1)	<0.01
Diabetes Mellitus						
Yes	17 (63.0)	0 (0.0)	<0.01	17 (68.0)	6 (24.0)	<0.01
No	68 (77.3)	3 (3.4)	<0.01	64 (71.1)	16 (17.8)	<0.01

Table 3 shows the percentage of patients in each group who had severe dysphagia before and after the therapy, along with the difference between the two measurements. The results indicate that both groups experienced a significant improvement in dysphagia after the therapy, as evidenced by the significant negative differences in the percentage of severe dysphagia before and after the therapy. However, the group that received behavioral therapy had a much greater improvement than the group that received normal therapy, as evidenced by the larger negative difference (-71.3% vs. -51.3%) and the lower percentage of severe dysphagia after the therapy (2.6% vs. 19.1%). The difference between the before and after measurements was statistically significant (P <0.01). The difference in the difference between the two groups was -20% (P<0.01), which means that the improvement in dysphagia is significantly greater in the group that received behavioral therapy compared to the group that received normal therapy (P<0.01).

**Table 3:** The effect of behavioral therapy on dysphagia of acute ischemic stroke patients.

	% of severe dysphagia			
	After	Before	Difference	P value
Behavioral therapy (n = 115)	2.6	73.9	-71.3	<0.01
Normal therapy (n = 115)	19.1	70.4	-51.3	<0.01
Difference	-16.5	3.5	-20.0	<0.01

Table 4 shows that behavioral therapy significantly reduced the percentage of severe dysphagia in acute ischemic stroke patients. The reduction percentage ranged from 18.3% to 21.6%, depending on the factors considered, such as sex, age, area, education, obesity, communication disorder, previous stroke, hypertension, and diabetes mellitus. When all factors were combined, they resulted in the highest reduction of 21.6% (all P<0.01). Negative values indicate that the therapy was effective in reducing severe dysphagia.

**Table 4:** The adjusted effect of behavioral therapy on dysphagia of acute ischemic stroke patients considering different factors.

Factor	% of severe dysphagia reduction adjusted by different factors	P value
Sex	-20.4	<0.01
Age	-19.7	<0.01
Area	-18.3	<0.01
Education	-20.1	<0.01
Obesity	-20.1	<0.01
Communication disorder	-20.3	<0.01
Previous stroke	-19.8	<0.01
Hypertension	-21.4	<0.01
Diabetes Melitus	-19.9	<0.01
All*	-21.6	<0.01

Note

*: Adjusted for sex, age, area, education, communication disorder, previous stroke, hypertension

## Discussion

This study represents a valuable addition to the field of stroke rehabilitation in Vietnam. It is the first research endeavor to investigate the effects of behavioral therapy on dysphagia patients with acute ischemic stroke who require nasogastric tube feeding. Our results unequivocally show that behavioral therapy produces better outcomes when combined with normal therapy. Specifically, we observed a remarkable 20% improvement in severe dysphagia, and the effectiveness of this intervention remained consistent even after controlling for various factors. These findings highlight the potential of behavioral therapy as an effective intervention for dysphagia patients.

The GUSS method was utilized in this study to assess dysphagia and grade severe cases. This method is particularly advantageous since it is straightforward and can be administered at the patient’s bedside [17]. Previous research has demonstrated the effectiveness of the GUSS method in evaluating the improvement of swallowing function in patients receiving pharyngeal electrical stimulation therapy [20], as well as in assessing dysphagia in COVID-19 patients admitted to the ICU [21], and in screening patients with acute ischemic stroke in Vietnam [22]. Additionally, the GUSS method has been utilized in studies examining the changes in dysphagia and nutritional status of patients with brain injury [23]. At present, the GUSS methodology has not been employed to assess the effects of behavioral therapy on dysphagia among patients with acute ischemic stroke, thereby impeding any potential comparisons with previous research studies.

The DID method is a widely recognized research design for evaluating the impact of an intervention on a specific outcome. While no studies have yet applied the DID method to estimate the effect of behavioral therapy on severe dysphagia, it remains a common approach [24–26]. The DID method involves comparing changes in outcomes between a treatment group and a control group before and after the intervention [27]. This approach accounts for any permanent differences between groups in unobservable factors that may affect outcomes, and attributes changes in the difference between group means after the intervention to the intervention itself [27]. Since this study was observational with a behavioral and a normal therapy group, the DID method is suitable for estimating the intervention’s effect.

Several studies were conducted to investigate the effects of behavioral interventions on swallowing function and outcomes in dysphagic patients. A discussed the translation of principles of neural plasticity into clinically oriented evidence for swallowing and dysphagia rehabilitation [28]. An updated Cochrane Review 2018 found that behavioral swallowing interventions significantly improved swallowing function in post-stroke dysphagia patients [29]. Furthermore, there was a trend for reducing the length of hospital stay and respiratory complications among dysphagia patients who used behavioral interventions [30].

Our study revealed a significant change in dysphagia levels before and after intervention in both the behavioral and normal therapy groups. This finding is consistent with previous studies documenting behavioral therapy’s positive effect on dysphagia levels [31]. Apart from behavioral therapy, other interventions were also found to be effective in improving patients’ dysphagia and quality of life. A randomized clinical trial by Langmore et al. revealed that electrical stimulation and exercise effectively improved dysphagia and quality of life in patients with head and neck cancer [32]. Similarly, a case series report by Eggmann et al. demonstrated that an intensive dysphagia rehabilitation program effectively reduced the severity of dysphagia and improved the quality of life in patients who had recovered from COVID-19 [33]. Furthermore, a randomized controlled trial by Krajczy et al. assessed the effects of dysphagia therapy in patients during the early post-stroke period. The study found that a comprehensive therapy for dysphagia effectively improved the swallowing reflex and reduced complications of swallowing disorders [34].

This study utilized a comprehensive behavioral therapy approach, incorporating various exercises to target the tongue, palate, facial muscles, and mouth movements. In a randomized controlled trial conducted by Choi et al., the effect of tongue-to-palate resistance training (TPRT) on tongue strength and oropharyngeal swallowing function in subacute stroke survivors with dysphagia was investigated [35]. The study concluded that TPRT effectively increased tongue muscle strength and improved swallowing function in these patients [35]. Tongue stretching exercises also enhanced tongue motility and oromotor function in stroke patients with dysphagia [36]. Isometric lingual exercises, which involved compressing an air-filled bulb between the tongue and the hard palate, effectively improved swallowing function [37]. Additionally, facial exercises were shown to play a significant role in addressing dysphagia in acute ischemic stroke patients [38]. Another study also suggested that exercises focused on the pharynx and larynx could enhance swallowing function [39].

It is important to acknowledge the limitations of any study, and this one is no exception. It should be noted that this was a single-center study, which means that our findings may not apply to other centers. In addition, the sample size was relatively small, which increases the possibility of type II error. Videofluoroscopic swallowing studies (VFSS) are widely recognized as the gold standard for evaluating swallowing function and identifying aspiration; however, we could not use VFSS in this study due to unfavorable conditions. Thus, using the GUSS method alone to assess swallowing function might result in overestimating the prevalence of dysphagia. We identified several more specific approaches for screening dysphagia [40], which could be considered for future study in the Vietnamese context. Additionally, stroke-associated pneumonia is a factor closely related to dysphagia and influences the prognosis of patients with acute ischemic stroke; however, the small sample size limited our ability to explore this issue in depth [41, 42]. One of the further limitations of this study is that we only selected patients with moderate strokes classified by NIHSS, so the results of this study cannot be generalized to stroke cases of other degrees. As this was an observational study, there may be variables we did not account for that could affect the results. Finally, the study did not assess the long-term effects of behavioral therapy on dysphagia, which needs to be investigated further.

## Conclusion

According to the study, incorporating behavioral therapy into standard post-stroke rehabilitation plans can improve swallowing function and reduce aspiration in individuals with acute ischemic stroke who undergo nasogastric tube feeding. Along with providing patients with appropriate dietary guidance, healthcare professionals should consider including behavioral therapy sessions in their rehabilitation strategies. To ensure the generalizability of these findings, further studies are needed to evaluate the long-term effects of behavioral therapy on dysphagia in acute ischemic stroke patients. Large multicenter research can help validate these results. Despite these limitations, the study demonstrates the promising potential of behavioral therapy as an intervention for dysphagia in acute ischemic stroke patients. In light of these findings, healthcare providers are encouraged to implement behavioral therapy as part of their post-stroke rehabilitation protocols.

## Supporting information

S1 Dataset (XLS)
